# Management of a pediatric patient with spontaneous cerebrospinal fluid leak in the lateral recess of sphenoid sinus and meningoencephalocele: A case report and literature review

**DOI:** 10.1016/j.ijscr.2023.108727

**Published:** 2023-08-29

**Authors:** Mashael AlKandery, Lulwah AlSaidan, Farhan Owayed, Marwan AlQunaee, Mutlaq Al-Sihan, Abdulmohsen Alterki

**Affiliations:** a Department of Otolaryngology, Head and Neck Surgery, Zain Hospital, Kuwait; b Department of Otolaryngology, Head and Neck Surgery, Zain Hospital, Shuwaikh Medical Area, Kuwait

**Keywords:** Cerebrospinal fluid leak, Lateral recess of sphenoid, Pediatric

## Abstract

**Introduction and importance:**

Spontaneous cerebrospinal fluid (CSF) leak in the lateral recess of the sphenoid (LRS) sinus in a pediatric population is rare and is surgically challenging to repair.

**Case presentation:**

We report a case of a 13-year-old girl with hydrocephalus and a ventriculoperitoneal shunt who presented with a two-month history of clear rhinorrhea from the right nostril. Computed tomography (CT) of the head was performed and showed CSF leak through a defect in the lateral recess of the sphenoid sinus. Defect closure was achieved using an endoscopic endonasal approach.

**Clinical discussion:**

CSF leak with meningoencephalocele from the sphenoid sinus is amongst the most difficult cases for repair. Yet the successful rate of repair is as high as 90 % if done endoscopically. Moreover, the location of the defect determines the surgical approach.

**Conclusion:**

Spontaneous CSF rhinorrhea from the lateral recess of the sphenoid (LRS) sinus, although rare, requires prompt diagnosis and treatment, as it may lead to significant mortality and morbidity.

## Highlights

• Spontaneous cerebrospinal fluid (CSF) rhinorrhoea is a rare condition leading to significant mortality and morbidity if not diagnosed and treated promptly.
• The success rate of endoscopic repair is as high as 90 % endoscopically, however the location of the defect determines the surgical approach.
• The incidence of meningoencephaloceles is 1 in 4000–5000 and tends to present as spontaneous CSF leaks

## 1 Introduction

Spontaneous cerebrospinal fluid (CSF) rhinorrhoea is a rare condition leading to significant mortality and morbidity if not diagnosed and treated promptly. This condition is caused by the disruption of the barriers between the anterior and middle cranial fossa and the sinonasal cavity. There are many different causes that could lead to this condition, which can be classified as either traumatic or non-traumatic [1]. In comparison with other locations, CSF leaks in lateral recess of sphenoid (LRS) sinus is a rare source of leak and imposes a surgical challenge during its repair [2]. Such cases may be managed via an endoscopic transphenoidal or transpterygoid approach [3].

Herein, we review our recent experience with a surgical repair in a pediatric patient that presented to our clinic with a spontaneous CSF leak in the LRS sinus.

This work has been reported in line with the SCARE 2020 criteria [4].

## 2 Presentation of case

**Fig. 1:**
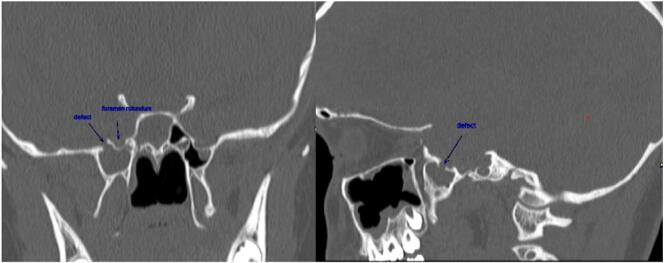
CT scan coronal and sagittal view showing meningoencephalocele through a defect in the roof of the right LRS sinus.

A 13 year-old female with hydrocephalus and a ventriculoperitoneal (VP) shunt that was inserted 6 months prior to her presenting complaint, presented to us with spontaneous clear fluid discharge from the right nostril of two months duration. There was no previous history of trauma, fever, chronic rhinitis, sinusitis or any previous surgical procedures. The patient did not complain of any headache, visual disturbances or nasal obstruction. Nasal endoscopy showed right-sided clear rhinorrhea with no oedema or other incidental findings. CT head revealed CSF leak through the defect in the right wall of the sphenoid sinus. Incidental marked bilateral hydrocephalus was found with J-shaped flattened sella and a scalloped inner calvarial cortex. (Fig. 1).

**Fig. 2:**
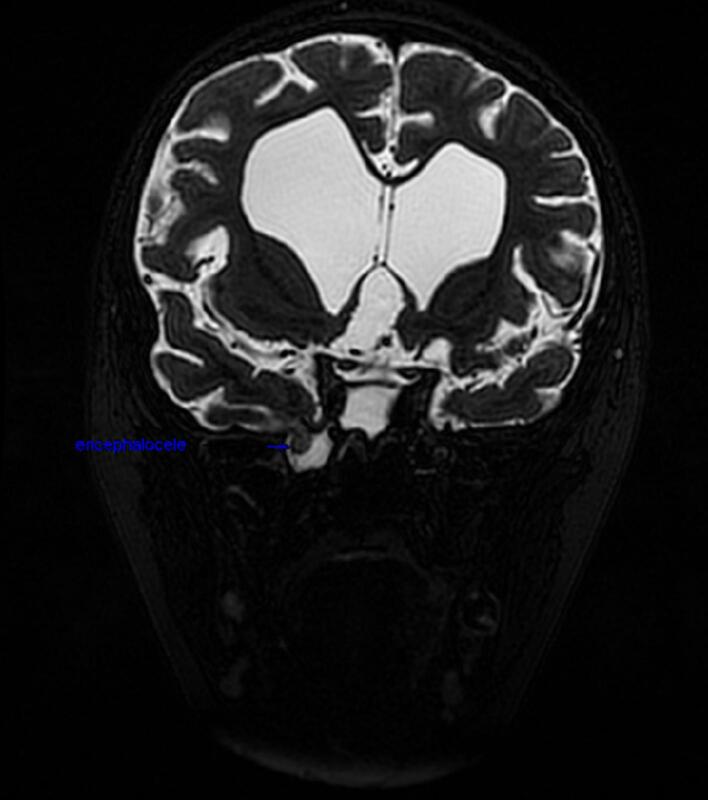
Bilateral hydrocephalus was found with J-shaped flattened sella and a scalloped inner calvarial cortex.

After which, MRI brain was done and confirmed the CSF leak and a tectal glioma causing aqueductal stenosis with moderate supraventricular hydrocephalus. A meningoencephalocele was also noted through a defect in the roof of the right LRS sinus. (Fig. 2).

The patient was taken for surgical repair by an otolaryngologist. Under general anaesthesia, an endoscopic endonasal approach was performed. Right sided uncinectomy, complete anterior and posterior ethmoidectomy and sphenoidotomy was done. Partial removal of the superior turbinate and lateral widening of the sphenoid ostium was performed. Portion of the medial pterygoid bone was removed with a Kerrison rounger for better access and visualization of the lateral extension of the sphenoid sinus. The defect was visualized lateral to the vidian canal, showing a high flow porous leak from the bone in the lateral recess of the sphenoid sinus.

Closure of the defect was done in a multilayer fashion using a tissue patch, followed by fat harvested from the paraumbilical area, fibrin sealant (Tisseel) then absorbable hemostatic agents (Surgicel) was applied to pack the repaired area. Intra-operatively, no leak was visualized following the repair. A merocel pack was inserted at the end of the procedure.

Post-operatively, the patient received intravenous ceftriaxone once daily, and was instructed complete bed rest with 10–15 degrees bed elevation for one-week duration. She was advised to avoid straining and lifting heavy objects for one month. Oral laxatives were prescribed up to three times a day. Subsequently, she was shifted to a neurosurgical center for close observation and active neurosurgical measures were taken to reduce her ICP. She had the lumbar drain removed, and a ventriculoperitoneal shut inserted successfully. Routine post-operative follow-ups up to one year revealed no CSF leak.

## 3 Discussion

The sphenoid sinus anatomy is highly variable within the population, and various degree of pneumatization of the LRS has been reported in 16–54 % of adults [3]. A spontaneous leak in the LRS is attributed to a congenital defect of Sternberg's canal, which may be exacerbated by meningiomas and raised intracranial pressure (ICP) [5]. As a result, an increase in pneumatization in the LRS leads to bony erosion and predisposes to meningoencephaloceles of the middle cranial fossa. Therefore, intraoperative risks are exceedingly high whilst performing CSF repairs due to bony dehiscence of the sphenoid sinus and the close proximity to adjacent neurovascular structures.

The sphenoid sinus (SS) is a paired paranasal sinus occurring within the body of the sphenoid bone, surrounded by important neurovascular structures. The SS invaginates into the posterior region of the nasal capsule at the sphenoethmoidal recess during the third month of intrauterine life [6]. There is no primary pneumatization, but rather a constriction of the developing pre-sphenoid recess followed by secondary pneumatization. Studies have shown that initial pneumatization of the sphenoid sinus varies from 6 months to 4 years of life [7]. Fifty percent of children are pneumatized by two years of age, 90 % show pneumatization by four years of age, and 100 % by ten years of age [8]. Its pneumatization pattern differs in different races, ethnic groups, and geographical locations. The types of pneumatization of the SS vary greatly in individuals, and are subdivided into conchal, lateral recess, presellar, and sellar, which can be complete or incomplete [9].

Patients tend to present with intermittent clear rhinorrhoea as their chief complaint. Other symptoms may include headaches, recurrent meningitis and seizures [3,13]. Therefore, diagnosing such cases requires a high index of suspicion. Initial diagnosis would be performing a Beta 2-transferrin test, which is considered a gold standard investigation with a sensitivity of 100 % and a specificity of 71 % [10]. In addition, non-invasive imaging modalities such as CT and MR Cisternography are crucial to accurately identify the site of leak. Furthermore, CSF leak from the sphenoid sinus is to be suspected whenever fluid gushes with a forward tilt of the head (positive Tea-pot sign) [12].

In several cases, for instance, posttraumatic CSF leaks may resolve with conservative measures such as bed rest, head elevation, avoidance of strenuous activities and reducing CSF pressure by a spinal tap or drain for 7–10 days. Leaks that persist despite conservative measures require surgical intervention to avoid serious life-threatening complications [12].

There have been various viewpoints in the literature with regards to the commonest locations for spontaneous CSF leaks. Although rare, some studies reported the incidence of a LRS sinus leak as high as 35 % [10]. Those cases were considered as the most surgically challenging cases to manage due to difficult endoscopic visualization and higher chances of postoperative recurrences.

CSF leak with meningoencephalocele from the sphenoid sinus is amongst the most difficult cases for repair [10]. While many cases reported in the literature claimed that a successful rate of repair is as high as 90 % endoscopically, the location of the defect determines the surgical approach. If the CSF leak occurs in the sphenoid near the midline, standard transnasal endoscopic approaches are usually adequate for closure. However, when there is a widely pneumatized sphenoid sinus extending into LRS, transpterygoid approach has shown to provide a wider corridor for access and successful repair. It is considered the most favorable approach with excellent outcomes in cases where the defect cannot be visualized properly [11,14]. The knowledge about surgical corridors for the approach to skull base is necessary when dealing with such cases, aiding in proper pre-operative planning [19].

After drilling the medial pterygoid plate, the vidian nerve may be identified and preserved by exposing it and limiting the drilling to the inferior portion of the medial pterygoid plate. Although the vidian nerve may be sacrificed, it can be useful to identify the internal carotid artery when followed posteriorly [18]. In cases where it is difficult to cover the LRS defect, obliteration of the sphenoid sinus or the lateral recess is recommended [11,12].

A variety of graft materials can be used in repair of CSF leaks depending on the size, site and the flow rate. However, superiority of one over the other has not been clearly established in the literature. Multilayered techniques in general have shown to yield optimal results [12].

Congenital skull base defects in pediatrics are rare and infrequently diagnosed [13,16]. The incidence of meningoencephaloceles is 1 in 4000–5000 and tends to present as spontaneous CSF leaks [13]. Encephaloceles occur due to abdnormal embryological layers development, leading to a persistent communication between the neuroectoderm and surface ectoderm. Ali Almomen et al. addressed various locations of skull base defects in pediatrics that were successfully repaired using an endoscopic endonasal approach [13]. Several authors consolidate that meticulous planning along with excellent surgical skills is paramount for successful repairs in such cases [15,16]. Furthermore, anatomic considerations such as the nasal pyriform aperture, sphenoid sinus pneumatization, and intercarotid distance within the sphenoid should be noted pre-operatively as it may limit surgical outcomes and options for reconstructive techniques [17].

Repair of defects in the LRS sinus in pediatric patients has not been widely discussed in the literature. In our case, we repaired the defect by an endoscopic transphenoidal approach using 0, 30 and 70-degree scopes, followed by a multilayered closure. The porous bony defect along with a high flow of CSF leak seen intraoperatively posed a challenge during surgical repair since there was no clear osteodural defect to patch. Moreover, given our patient's history of raised ICP and a spontaneous ‘high flow’ CSF leak, a temporary lumbar drain was used as a CSF diversion technique [10]. A permanent VP shunt to avoid the failure of repair was subsequently placed by neurosurgeons.

## 4 Conclusion

Spontaneous CSF rhinorrhea from the lateral recess of the sphenoid (LRS) sinus, although rare, is surgically challenging. The aim of this paper was to report our experience with CSF leak and meningoencephalocele in a pediatric patient that was repaired successfully using an endoscopic endonasal approach.

## Ethical approval

Not declared.

## Funding

This research did not receive any specific grant from funding agencies in the public, commercial, or not-for-profit sectors.

## CRediT authorship contribution statement

Dr. Mashael AlKandery; data collection, data analysis, and write up.

Dr. Lulwah AlSaidan; data collection, data analysis, and write up.

Dr. Farhan Owayed; data collection, data analysis, and write up.

Dr. Marwan AlQunaee; data analysis and contribution.

Dr. Mutlaq Al-Sihan; data analysis and contribution.

Dr. Abdulmohsen AlTerki; data analysis and contribution.

## Guarantor

Dr. Lulwah AlSaidan

## Parental consent (for minors)

Written informed consent was obtained from the patient's parents/legal guardian for publication and any accompanying images. A copy of the written consent is available for review by the Editor-in-Chief of this journal on request.

## Declaration of competing interest

There is no conflict of interest to declare by any of the authors of this study.

## References

[1] Keshri Amit (2019). Management of spontaneous CSF rhinorrhea: an institutional experience. J. Neurol. Surg. B Skull Base.

[2] Alexander Nathan S. (2012). Treatment strategies for lateral sphenoid sinus recess cerebrospinal fluid leaks. Arch. Otolaryngol. Head Neck Surg..

[3] Maxfield Alice Z. (2020). Endoscopic management of lateral sphenoid cerebrospinal fluid leaks: identifying a radiographic parameter for surgical planning. Laryngoscope Invest. Otolaryngol..

[4] Agha R.A., Franchi T., Sohrabi C., Mathew G., for the SCARE Group (2020). The SCARE 2020 Guideline: updating consensus Surgical CAse REport (SCARE) guidelines. Int. J. Surg..

[5] Mona Moudi Merlin, Sutikno Budi (2021). Management of cerebrospinal fluid leak in the lateral recess of the sphenoid sinus with transpterygoid approach: a case report. Int. J. Surg. Case Rep..

[6] Kim H.S., Park E.K., Choi P.C., Chung H.Y., Kim J.N., Chung S.P. (1993). Normal development of the paranasal sinuses in children: a CT study. Daehan Bangsa’seon Yihag Hoeji.

[7] Jang Y.H., Kim S.U. (2000). Pneumatization of the sphenoid sinus in children evaluated by magnetic resonance imaging. Am. J. Rhinol..

[8] Fujioka M., Young L.W. (1978). The sphenoidal sinuses: radiographic patterns of normal development and abnormal findings in infants and children. Radiology.

[9] Hiremath S.B., Gautam A.A., Sheeja K., Benjamin G. (2018). Assessment of variations in sphenoid sinus pneumatization in Indian population: a multidetector computed tomography study. Indian J. Radiol. Imag..

[10] Keshri Amit (2019). Management of Spontaneous CSF rhinorrhea: an institutional experience. J. Neurol. Surg. B Skull Base.

[11] Babu A.R. (2019). Contrasting surgical management of CSF leak from lateral recess of sphenoid sinus and its surgical outcomes: our experience. Indian J. Otolaryngol. Head Neck Surg..

[12] Janakiram Trichy Narayanan (2015). Endoscopic Endonasal repair of sphenoid sinus cerebrospinal fluid leaks: our experience. Indian J. Otolaryngol. Head Neck Surg..

[13] Al Momen Ali (2021). The endonasal endoscopic management of pediatric CSF leak and meningoceles. Am. J. Otolaryngol. Head Neck Surg..

[14] Zhou Bing (2007). Endoscopic transpterygoid intervention of meningoencephalocele within lateral recess of sphenoid. Chin. J. Otorhinolaryngol. Head Neck Surg..

[15] Nation Javan (2018). Pediatric endoscopic endonasal approaches for skull base lesions in the very young: is it safe and effective?. J. Neurol. Surg. B Skull Base.

[16] Castelnuovo Paolo (2009). Endoscopic endonasal management of encephaloceles in children: an eight-year experience. Int. J. Pediatr. Otorhinolaryngol..

[17] Stapleton Amanda L. (2017). Risk factors for cerebrospinal fluid leak in pediatric patients undergoing endoscopic endonasal skull base surgery. Int. J. Pediatr. Otorhinolaryngol..

[18] Schmidt Richard F. (2012). Surgical nuances for the endoscopic endonasal transpterygoid approach to lateral sphenoid sinus encephaloceles. Neurosurg. Focus..

[19] Sireci F., Danè G. (2021). Dispenza F et al Main corridors in the transphenoidal skull base surgery. Nova publisher. Adv. Health Dis..

